# Does the common sexually transmitted parasite *Trichomonas vaginalis* have sex?

**DOI:** 10.1371/journal.ppat.1006831

**Published:** 2018-03-08

**Authors:** Martina Bradic, Jane M. Carlton

**Affiliations:** Center for Genomics and Systems Biology, Department of Biology, New York University, New York, New York, United States of America; University of Wisconsin Medical School, UNITED STATES

## Introduction

The parasite *Trichomonas vaginalis* is a haploid, flagellated, eukaryotic microbe that adheres to the human urogenital tract and causes the most common sexually transmitted parasitic infection, trichomoniasis ("trich"), with around 250 million new cases reported annually worldwide and about 7 million cases in the United States [1]. Previously considered a female “nuisance disease,” *T*. *vaginalis* has now been associated with adverse pregnancy outcomes such as preterm delivery, low birth weight, increased risk of HIV infection, and cervical and prostate cancers [2]. In contrast to other parasitic protists that can encyst (e.g., *Giardia*), *T*. *vaginalis* appears to have only one morphological form, a sexually transmitted, mitotically dividing trophozoite. Besides its important role as the causative agent of trich, the parasite is of interest due to its unusually large genome size (around 160 Mb), of which 65% is made up of families of transposable elements (TEs), members of which are highly similar to each other [3]. The nature and size of the *T*. *vaginalis* genome raises questions about its evolution and, in particular, how this supposedly asexually reproducing organism survives the deleterious effects of so many active TE families. Indeed, several lines of evidence described below suggest that *T*. *vaginalis* may engage in genetic exchange or has done so in its recent evolutionary past. Elucidating a possible sexual cycle in *T*. *vaginalis* is crucial not only for learning about its biology and parasitism (e.g., virulence and spread of drug resistance), but also to provide generalizable models for the evolution of sex in other parasites.

## What is sex and why is it important for parasitism?

There has been a debate about sexual cycles in eukaryotic microbes for many years. Sexual reproduction is a process by which specialized reproductive cells fuse, contributing genetic information to produce unique progeny. It is considered a major source of genetic diversity in a population and thus advantageous because it accelerates adaptation to fluctuating environments or purges deleterious mutations. There is a cost to sex, however, such as the disruption of well-adapted combinations of alleles. In fact, asexual reproduction is predicted to be advantageous as a short-term evolutionary strategy under many conditions. In an asexual (clonal) population, all members of the population carry the same genetic information, and only mutations, horizontal gene transfer, or genome rearrangements contribute to genetic variation. Only a few fungi are thought to be truly asexual. Cryptic sex, on the other hand, including parasexual or unisexual reproduction, is a common reproductive strategy in fungi (e.g., the *Candida* species complex) and some parasitic protists [4]. In a parasexual cycle, two cells and their nuclei fuse followed by chromosome loss, resulting in cells that can vary in their final ploidy [5]. Alternatively, unisexual reproduction introduces more limited genetic diversity through mother–daughter cell–cell fusion or "endoreplication" and has been found in parasites such as *Giardia intestinalis* and *Leishmania* [6].

The success of parasites and the epidemiology of the diseases they cause is directly associated with their capacity to produce genetically variable infections. Sexually reproducing parasites create genetic variation through recombination (by merging genetic material from different parasites), thereby creating new combinations of genes that the parasite can use to overcome the host immune system or to develop drug resistance [7]. Thus, identifying the mode of reproduction of a parasite is important to determine how it will spread and how to treat it.

## Population genetic evidence for sex in *T*. *vaginalis*

**Fig 1:**
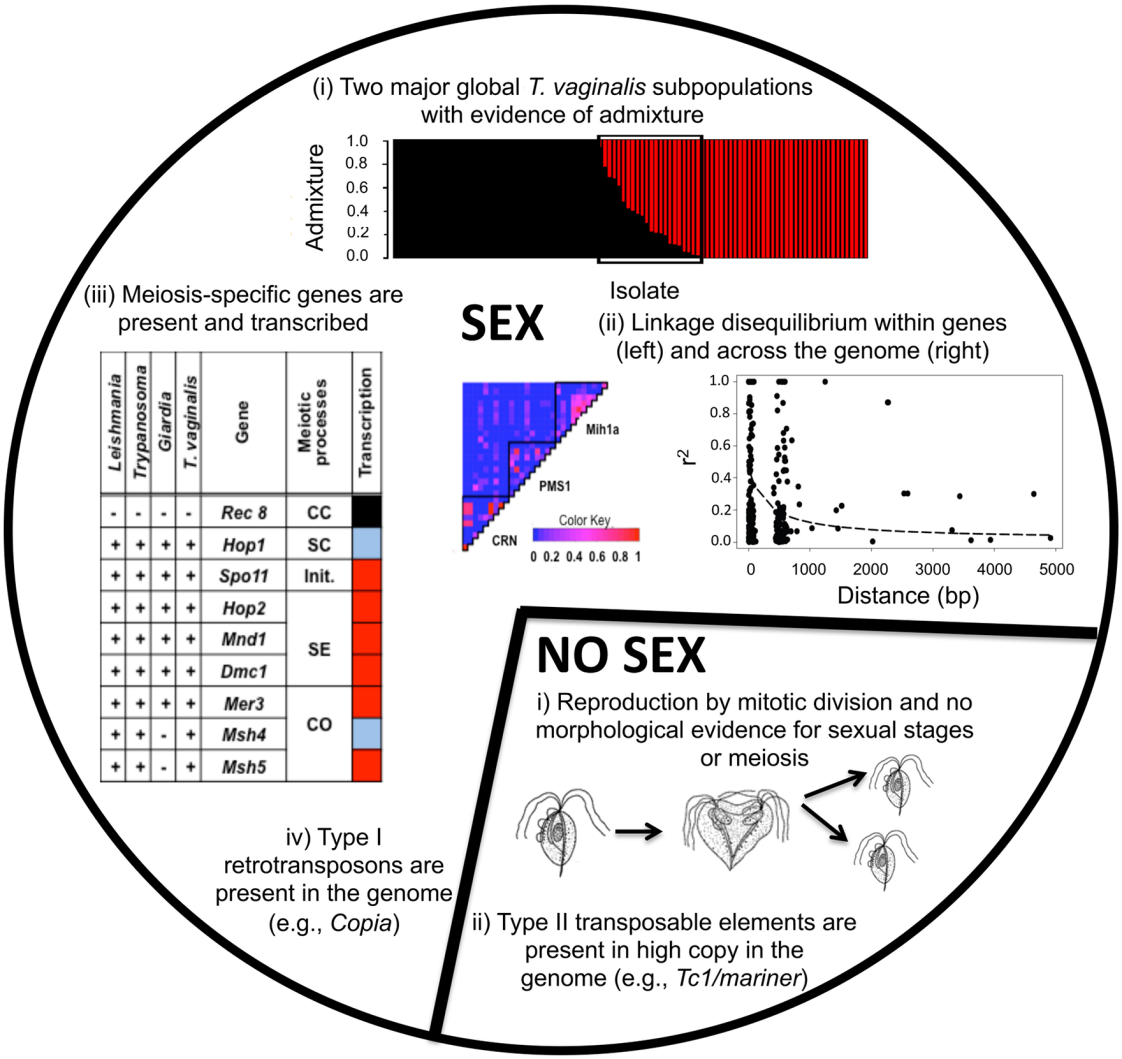
Summary of evidence suggesting sexual or asexual reproduction in *T*. *vaginalis*. The large slice (SEX) presents evidence for sexual reproduction: (i) Population admixture plot based on analysis of 3,923 SNP markers in 102 global *T*. *vaginalis* isolates (data from [13]). Each column represents a single *T*. *vaginalis* isolate and shows the proportion of its genotype as a part of each of two subpopulations (black and red). Isolates containing both black and red genotypes represent potential recombinants between the two subpopulations. (ii) LD decays fast within genes [10], as well as across the genome [13]. A heat map represents the degree of LD between 49 SNPs in three single-copy genes, with r^2^ (the standardized measure of LD between pairs of SNPs) colored according to low LD (0; blue) and high LD (1; red); black lines indicate gene boundaries. The graph shows LD decay (r^2^, y-axis) calculated over 5-kb intervals (distance, x-axis) in 2,837 SNPs from 872 *T*. *vaginalis* contigs. Each point represents the average LD between two SNPs that are 5 kb apart. (iii) Homologs of nine major meiosis-specific genes are present in *T*. *vaginalis* and other sexually reproducing protozoan parasites [15]. Transcription of eight of these genes (excluding Rec8 [black square]) is detectable at levels above (red squares) or below (blue squares) the average expression of all *T*. *vaginalis* genes (RNA next generation sequencing [RNA-seq] data from [13]). (iv) The presence of Type I retro TEs (e.g., Copia, a family of long terminal repeat elements that move by means of an RNA intermediate, common in animals, fungi, protista, and plants), suggests sexual recombination in *T*. *vaginalis*. The small slice (NO SEX) presents evidence for asexual reproduction: (i) No visible sexual stages have been identified for *T*. *vaginalis* under any conditions tested. (ii) A high abundance of extremely similar Type II TEs (e.g., Tc1/mariner, a family of transposons found throughout metazoa that use a cut-and-paste mechanism to transpose) implies their accumulation due to a lack of sexual reproduction. CC, cohesin complex; CO, crossover; Init., initiation of double-strand break; LD, linkage disequilibrium; SC, synaptonemal complex; SE, strand exchange; SNPs, single nucleotide polymorphisms.

The genetic diversity of a parasite population and linkage disequilibrium (LD), the nonrandom segregation of alleles at different loci in a population, represent powerful metrics by which to evaluate evidence for sexual reproduction of an organism. Population genetics theory predicts that clonally reproducing organisms show low genetic diversity and high LD [8], whereas sexually reproducing organisms show high genetic diversity, population structuring, and independent segregation of alleles (linkage equilibrium) as a result of recombination. Several studies using a variety of genetic markers, including multi-locus strain typing (MLST) [9], microsatellites [10, 11], TE insertion polymorphisms [12], and single nucleotide polymorphisms (SNPs) [13, 14] have been used on global sets of *T*. *vaginalis* isolates to determine whether the parasite follows population genetic trends consistent with asexual reproduction. These studies have revealed high genetic diversity of *T*. *vaginalis* parasites and the presence of two global parasite subpopulations (Fig 1). Moreover, LD has been identified both within genes [10] and across the *T*. *vaginalis* genome (LD decay within 5 kb [13]), a pattern representative of frequently recombining organisms consistent with sexual reproduction. The rate of decay in LD is also a good indicator of recombination rates in the population. Our studies demonstrated faster LD decay and thus a higher recombination rate in one subpopulation over the other [10, 13]. In addition, isolates harboring alleles from both subpopulations have been identified (Fig 1), which may represent recombinant parasites between the two subpopulations and suggest *T*. *vaginalis* admixture (interbreeding between two isolated populations within a species). Thus, *T*. *vaginalis* population genetics strongly supports the ability of the parasite to undergo some form of genetic exchange or suggests the parasite could do so at some stage during its evolutionary past.

## Molecular genetic evidence for sex in *T*. *vaginalis*

What are the lines of molecular genetic evidence for a sexual cycle in *T*. *vaginalis*? Malik et al. [15] mined the genome sequence to identify a nearly full complement of meiosis genes (27 of 29) in the *T*. *vaginalis* genome, suggesting that the parasite may be equipped to perform meiotic recombination or a similar parasexual process by using its meiotic gene homologs. Moreover, eight genes specific for meiosis in model organisms and known to exist mostly in sexually reproducing species were also present in *T*. *vaginalis*. Potential morphological evidence for recombination has also been described, such as multinucleated cells in *T*. *vaginalis* and “budding” in other species of trichomonads (indicating polyploidy and the potential for recombination [16]). We evaluated expression of the eight meiosis-specific genes (Spo11, Hop1, Hop2, Mnd1, Dmc1, Mer3, Msh4, Msh5) in *T*. *vaginalis* using RNA-seq from the reference *T*. *vaginalis* strain G3 [13]. None of the eight genes contains stop codons or nonsense mutations, and six of them were transcribed at levels above the average expression of all genes in *T*. *vaginalis*, suggesting meiosis to be an active process (Fig 1). Although the presence and transcription of meiosis-specific genes suggests the parasite has a sexual cycle, other parasitic protists (e.g., *G*. *intestinalis*, *Trypanosoma cruzi*) also contain meiosis genes but undergo a cryptic sexual cycle [17] or unisexual reproduction [6]. Further studies in *T*. *vaginalis* will be needed to learn about the function of the meiosis-specific genes.

## Presence of retrotransposons as evidence for sex in *T*. *vaginalis*

TEs are ubiquitous and present in many living organisms, and their type, diversity, and frequency of occurrence in a genome are some predictors of the type of reproduction. Asexual organisms frequently lack Class I TEs (also called retrotransposons, derived from RNA), while organisms that undergo a sexual cycle can contain Class II (DNA-derived) TEs as well as Class I TEs [18]. The *T*. *vaginalis* genome is unique among parasitic protists because it contains 30,000–40,000 Class I and II TEs in more than 50 different families, with TEs making up close to 40% of the genome. In addition, there are far fewer Type I than Type II TEs, and members of each family show very little genetic diversity [3]. Studies have shown that members of the Type II *Tc1/mariner* family are active [19], show insertion-site polymorphisms among different strains, and exhibit reduced expression of *T*. *vaginalis* genes in close proximity to a *mariner* insertion [12]. Thus, the deleterious effects of active and transposing TEs in the *T*. *vaginalis* haploid genome have the potential to be exceptionally high, especially if the parasite is asexual, and could potentially lead to its extinction. While *T*. *vaginalis* has most likely developed a mechanism to mitigate the deleterious effects of TEs, it seems most likely that stable TE copy numbers in *T*. *vaginalis* are maintained through the interplay of recombination, sexual reproduction, and natural selection, as has been hypothesized [20].

Sexual reproduction is highly common among eukaryotes, and many eukaryotic microbial pathogens have recently been found to have extant cryptic sexual cycles, enabling them to increase genetic diversity, purge deleterious mutations, and be successful in the face of host immunity or drug pressure. While we know that *T*. *vaginalis* is transmitted during sexual contact, we don't know whether the parasite itself has sex. Here we have summarized some of the recent advances in *T*. *vaginalis* biology that provide compelling evidence that the parasite has an active sexual cycle—possibly cryptic—or had sex recently in its evolutionary past.

## References

[1] World Health Organization. Prevalence and incidence of selected sexually transmitted infections, Chlamydia trachomatis, Neisseria gonorrhoeae, syphilis and Trichomonas vaginalis: methods and results used by WHO to generate 2005 estimates. 2011.

[2] Kissinger P. Trichomonas vaginalis: a review of epidemiologic, clinical and treatment issues. BMC infectious diseases. 2015;15:307 doi: 10.1186/s12879-015-1055-0 . 26242185 10.1186/s12879-015-1055-0 PMC4525749

[3] Carlton JM, Hirt RP, Silva JC, Delcher AL, Schatz M, Zhao Q, et al Draft genome sequence of the sexually transmitted pathogen Trichomonas vaginalis. Science. 2007;315(5809):207–12. Epub 2007/01/16. doi: 10.1126/science.1132894 . 17218520 10.1126/science.1132894 PMC2080659

[4] Heitman J. Evolution of eukaryotic microbial pathogens via covert sexual reproduction. Cell Host Microbe. 2010;8(1):86–99. doi: 10.1016/j.chom.2010.06.011 . 20638645 10.1016/j.chom.2010.06.011 PMC2916653

[5] Bennett RJ. The parasexual lifestyle of Candida albicans. Current opinion in microbiology. 2015;28:10–7. doi: 10.1016/j.mib.2015.06.017 . 26210747 10.1016/j.mib.2015.06.017 PMC4688137

[6] Feretzaki M, Heitman J. Unisexual reproduction drives evolution of eukaryotic microbial pathogens. PLoS Pathog. 2013;9(10):e1003674 doi: 10.1371/journal.ppat.1003674 . 24204257 10.1371/journal.ppat.1003674 PMC3814335

[7] McDonald BA, Linde C. Pathogen population genetics, evolutionary potential, and durable resistance. Annu Rev Phytopathol. 2002;40:349–79. doi: 10.1146/annurev.phyto.40.120501.101443 12147764 10.1146/annurev.phyto.40.120501.101443

[8] Schurko AM, Neiman M, Logsdon JM Jr., Signs of sex: what we know and how we know it. Trends in ecology & evolution. 2009;24(4):208–17. doi: 10.1016/j.tree.2008.11.010 . 19282047 10.1016/j.tree.2008.11.010

[9] Cornelius DC, Robinson DA, Muzny CA, Mena LA, Aanensen DM, Lushbaugh WB, et al Genetic characterization of Trichomonas vaginalis isolates by use of multilocus sequence typing. Journal of clinical microbiology. 2012;50(10):3293–300. doi: 10.1128/JCM.00643-12 . 22855512 10.1128/JCM.00643-12 PMC3457461

[10] Conrad MD, Gorman AW, Schillinger JA, Fiori PL, Arroyo R, Malla N, et al Extensive Genetic Diversity, Unique Population Structure and Evidence of Genetic Exchange in the Sexually Transmitted Parasite Trichomonas vaginalis. PLoS Negl Trop Dis. 2012;6(3). doi: 10.1371/journal.pntd.0001573 22479659 10.1371/journal.pntd.0001573 PMC3313929

[11] Prokopi M, Chatzitheodorou T, Ackers JP, Clark CG. A preliminary investigation of microsatellite-based genotyping in Trichomonas vaginalis. T Roy Soc Trop Med H. 2011;105(8):479–81. doi: 10.1016/J.Trstmh.2011.05.005 21700304 10.1016/j.trstmh.2011.05.005

[12] Bradic M, Warring SD, Low V, Carlton JM. The Tc1/mariner transposable element family shapes genetic variation and gene expression in the protist Trichomonas vaginalis. Mobile DNA-Uk. 2014;5 doi: 10.1186/1759-8753-5-12 24834134 10.1186/1759-8753-5-12 PMC4021607

[13] Bradic M, Warring SD, Tooley GE, Scheid P, Secor WE, Land KM, et al Genetic indicators of drug resistance in the highly repetitive genome of Trichomonas vaginalis. Genome Biol Evol. 2017;9(6):1658–72. doi: 10.1093/gbe/evx110 . 28633446 10.1093/gbe/evx110 PMC5522705

[14] Paulish-Miller TE, Augostini P, Schuyler JA, Smith WL, Mordechai E, Adelson ME, et al Trichomonas vaginalis metronidazole resistance is associated with single nucleotide polymorphisms in the nitroreductase genes ntr4Tv and ntr6Tv. Antimicrobial agents and chemotherapy. 2014;58(5):2938–43. doi: 10.1128/AAC.02370-13 . 24550324 10.1128/AAC.02370-13 PMC3993245

[15] Malik SB, Pightling AW, Stefaniak LM, Schurko AM, Logsdon JM Jr. An expanded inventory of conserved meiotic genes provides evidence for sex in Trichomonas vaginalis. PLoS ONE. 2008;3(8):e2879 doi: 10.1371/journal.pone.0002879 . 18663385 10.1371/journal.pone.0002879 PMC2488364

[16] Pereira-Neves A, Benchimol M. Tritrichomonas foetus: budding from multinucleated pseudocysts. Protist. 2009;160(4):536–51. doi: 10.1016/j.protis.2009.05.001 . 19616999 10.1016/j.protis.2009.05.001

[17] Ramesh MA, Malik SB, Logsdon JM Jr., A phylogenomic inventory of meiotic genes; evidence for sex in Giardia and an early eukaryotic origin of meiosis. Current biology: CB. 2005;15(2):185–91. doi: 10.1016/j.cub.2005.01.003 . 15668177 10.1016/j.cub.2005.01.003

[18] Arkhipova I, Meselson M. Transposable elements in sexual and ancient asexual taxa. Proceedings of the National Academy of Sciences of the United States of America. 2000;97(26):14473–7. doi: 10.1073/pnas.97.26.14473 . 11121049 10.1073/pnas.97.26.14473 PMC18943

[19] Silva JC, Bastida F, Bidwell SL, Johnson PJ, Carlton JM. A potentially functional mariner transposable element in the protist Trichomonas vaginalis. Mol Biol Evol. 2005;22(1):126–34. doi: 10.1093/molbev/msh260 15371525 10.1093/molbev/msh260 PMC1406841

[20] Dolgin ES, Charlesworth B. The fate of transposable elements in asexual populations. Genetics. 2006;174(2):817–27. doi: 10.1534/genetics.106.060434 . 16888330 10.1534/genetics.106.060434 PMC1602064

